# Advances in precision neuromodulation: electroconvulsive therapy amplitude titration

**DOI:** 10.1038/s41386-024-01797-3

**Published:** 2024-01-09

**Authors:** Paul E. Croarkin, Alexander Opitz

**Affiliations:** 1 Mayo Clinic Department of Psychiatry and Psychology, Rochester, MN USA; 2 Department of Biomedical Engineering, University of Minnesota, Minneapolis, MN USA

**Keywords:** Translational research, Neurophysiology

Electroconvulsive therapy (ECT) stands as the oldest and most effective treatment modality used in psychiatry. With over 80 years of research efforts and clinical experience, there have been considerable advances in the delivery of ECT. However, there are ongoing challenges for patients, clinical practice, and unanswered research questions. The cognitive side effect profile of ECT contributes to patient burden, barriers to access, early discontinuation of therapy, and unfortunate stigma surrounding this life-saving treatment. Modern research efforts focus on innovations that may improve the cognitive side effect burden of ECT while maximizing clinical efficacy.

**Fig. 1:**
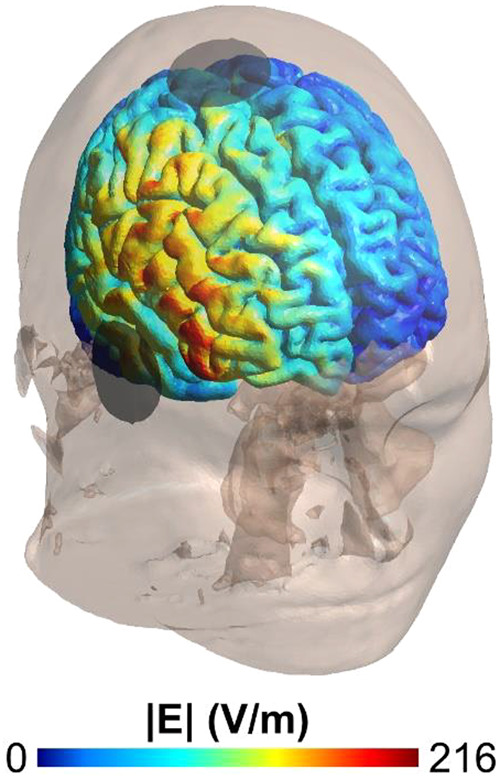
ECT electric field distribution [V/m] in the brain for a RUL montage and 800 mA stimulation amplitude.

Knowledge gaps related to the therapeutic mechanism of ECT provide challenges and opportunities, for these efforts. It is not known if customizing the delivery of electrical stimulation, modulating the induced seizure, or both will impact clinical efficacy and tolerability. Current clinical ECT delivers nonfocal and strong electric fields (E-fields) yielding generalized seizures (Fig. 1). Promising prior and ongoing efforts focus on refining the E-field delivery and ensuing seizures include work with magnetic seizure therapy, low amplitude electric stimulation, focal electrically administered seizure therapy, frontomedial ECT, and electric (E-field) modeling [1].

Other work has refined the stimulus parameters of ECT such as electrode placement, temporal waveform, pulse width, pulse shape, train frequency, train duration, and train directionality. Pulse amplitude is a principal aspect of electric field intensity and focality. In contemporary ECT practice, pulse amplitude is arbitrarily set at 800 or 900 milliamperes (mA) which produces E-field strengths that surpasses activation thresholds of most neuronal pathways and exceeds what is necessary to generate a seizure for therapeutic purposes. Until recently, surprisingly little research has focused on individualized or variable amplitude dosing for ECT. A rigorous line of preclinical and translational efforts focused on amplitude dosing has developed in *Neuropsychopharmacology* and elsewhere [2]. E-field strength is associated with improved clinical efficacy yet carries a greater neurocognitive burden. Titration approaches examining amplitude and E-field strength may present opportunities to identify ECT dosing that ensures antidepressant effects with minimal neurocognitive burden [3].

Abbott and colleagues now examine clinical antidepressant and neurocognitive outcomes with an amplitude titration method guided by E-field modeling. Older patients (aged 50–80 years) undergoing ECT underwent amplitude-based seizure titration. Stimulation commenced at 100 mA, increasing in 70 mA increments thereafter every 30 s until a seizure was elicited. The remaining treatments were delivered with right unilateral electrode (RUL) placement and 800 mA. A 800 mA/amplitude seizure threshold (STa) established the relative dose. Clinical antidepressant, neurocognitive outcome measures, and right hippocampal volume changes were monitored. While findings have not been consistently replicated, prior work suggests that right hippocampal E-field strength is directly related to neurocognitive outcomes and may impact clinical efficacy. In the present study, treatments with greater STa yielded less clinical improvement in depressive symptom severity. Greater whole brain E-field (but not STa) was associated with decreased category fluency performance. Right hippocampal volume increases were noted earlier with lower STa treatment. Whole brain E-field and STa had considerable variability among patients challenging current fixed amplitude dosing [4]. The present findings underscore the importance of STa and whole brain E-field relationships. Notwithstanding limitations, the study is an important step forward for precision dosing of ECT.

There are limitations and pragmatic considerations for future clinical translations of current amplitude seizure threshold titration and dosing of ECT. The study had a relatively small sample size, focused on right unilateral electrode placement, and the titration utilized a somewhat broad amplitude titration approach (70 mA increments). The E-field computational approach made the necessary assumption that tissue conductivities were the same across individuals. Also, temporal aspects of dosing were not considered in the computational approach which might be a promising line for future investigations. This present amplitude titration approach is also confined to a research environment. Available ECT devices do not allow for sufficient manipulation of current amplitude. The costs and efforts required to bring new devices or related technologies to market are considerable. The regulatory pathways for precision ECT devices are complex with related time and financial barriers. An anatomical magnetic resonance imaging scan and processing are requisite for E-field computational approaches. A recent study also suggested that ketamine treatments were clinically equivalent to ECT for the treatment of non-psychotic major depressive disorder. Emerging, rapid-acting antidepressants may have practical and economic advantages as compared to new generation ECT devices for patient centered treatments [5].

Irrespective of pragmatic considerations and limitations of the present findings, the study does underscore the importance of E-field modeling for future neuromodulation interventions and research. Despite considerable prior efforts, dosing strategies for ECT and other brain stimulation interventions are not well defined. Neurophysiological, neuroanatomical, and the neural underpinnings of psychiatric illnesses present complex heterogeneity for therapeutic interventions. Individualized E-field dosing provides a framework for standardization of treatment in study decision and clinical practice. Predictions and refinement of E-field distributions facilitates precision and effectiveness for therapeutic brain stimulation therapies. Computational work adds information regarding the tissue conductivity, geometry, and electrode placement or targeting. These techniques will continue to inform understanding of dose-response, classification of brain phenotypes, and innovations for novel therapeutics.

This study advances understanding and opportunities for decision making algorithms for dosing ECT. Current ECT practice centers around general empiric approaches to minimize cognitive risk, counterbalanced by choices that increase clinical efficacy such as dosing with bitemporal electrodes. Amplitude individualizations consider individual neuroanatomy and brain electric field with the goal of rapid treatment response and minimal neurocognitive burden.
